# GLA-3 mediates the heat shock response in *Caenorhabditis elegans* germ cells: A key role for the tristetraprolin (TTP) family

**DOI:** 10.1371/journal.pone.0312069

**Published:** 2026-01-02

**Authors:** Laura Silvia Salinas, Ángel Armando Dámazo-Hernández, Arianne Melisa Cristino-Miranda, Mariana Zurita-León, Enrique Morales-Oliva, Laura Ivón Láscarez-Lagunas, Rosa Estela Navarro

**Affiliations:** Departamento de Biología Celular y Desarrollo, Instituto de Fisiología Celular, Universidad Nacional Autónoma de México, Mexico, Mexico; East Carolina University, UNITED STATES OF AMERICA

## Abstract

Tristetraprolin or TTP is an RNA-binding protein that possesses two CCCH-like zinc-finger domains that bind AU-rich elements to promote their degradation. One of its targets is the mRNA of tumor necrosis factor alpha (TNF-α). When TTP is absent, the TNF-α factor accumulates causing severe, generalized inflammation in knockout mice. TTP is also considered a tumor suppressor protein because it regulates the expression of several mRNAs that encode for proteins involved in cell cycle regulation and it is downregulated in various types of human cancers. Under stress, TTP associates with stress granules (SGs), dynamic cytoplasmic condensates formed by liquid-liquid phase separation (LLPS) that protect mRNAs from harmful conditions. Despite TTP’s important role in mRNA turnover, much remains to be explored about its participation in stress resistance in living animals. For this reason, we investigated the role of GLA-3, one of TTP’s homologs, in the nematode *Caenorhabditis elegans* during the heat shock response. Previously, it has been shown that nematodes lacking *gla-3*/TTP exhibit phenotypes such as progressive loss of motility, reduced brood size, and increased embryonic lethality. As well as defects in meiotic progression, and increased germ-cell apoptosis. Here, we show that a GFP::GLA-3 reporter is primarily expressed in the *C. elegans* germline. During heat shock, GLA-3 localizes to condensates that contain both processing bodies, sites of mRNA storage and decay, and stress granules. We demonstrate that, in the *C. elegans* gonad under heat shock conditions, the canonical P body marker CGH, the DDX6 homolog, associates with GLA-3, as well as with the canonic stress markers TIAR-1/TIA1 and GTBP-1/G3BP. These data show that in *C. elegans*, P bodies and stress granules colocalize during heat shock. Similarly, in yeast, P bodies and stress granules fuse during stress, suggesting that *C. elegans* induces condensates that resemble those observe in yeast. Additionally, we demonstrate that GLA-3 is important for the formation of both P bodies and stress granules. Finally, we show that oogenic germ cells of GLA-3 mutant animals that were exposed to heat shock resulted in embryos that did not survive, showing that GLA-3 plays an important role in protecting germ cells from this condition. Our results demonstrate that the role of GLA-3 is conserved in *C. elegans*, and this model can be very useful for further investigating the role of this protein in the future.

## Introduction

Germ cells transmit essential information for the next generation by providing maternal mRNA and RNA-binding proteins that regulate mRNA expression during early embryogenesis. Consequently, the control of translation is key in germline development and function. RNA granules or ribonucleoprotein complexes (RNPc) are biomolecular condensates formed by liquid-liquid phase separation (LLPS) that control mRNA regulation [1–3]. Stress granules (SGs) are among the best-studied biomolecular condensates; they are assembled mainly during the arrest of translation initiation triggered by stressful conditions [4]. RNA granule formation is orchestrated by proteins with intrinsically disordered regions (IDR) and/or low-complexity domains (LCD) [5]. Among the key proteins that trigger SG formation are RNA-binding proteins such as the T-cell-restricted intracellular antigen-1 protein (TIA-1/TIAR) [6] and Tristetraprolin (TTP) [7], which contain prion-like and IDR domains, respectively, and play essential roles in RNA granule nucleation [8–10].

The TTP family of proteins plays an important role in mRNA regulation. TTP is part of a family of CCCH tandem zinc finger proteins (TIS11) that interacts directly with the AU-rich elements (AREs) of mRNA 3’UTR [11] to promote deadenylation and, eventually, its degradation [12]. For example, TTP promotes the degradation of tumor necrosis factor alpha (TNF-α) and other cytokines mRNA, explaining why mutant mice lacking TTP have severe, generalized inflammation [11]. The lower expression of TTP has also been related to cancer; thus, this protein is considered a tumor suppressor in many types of cancer [13].

Under non-stressful conditions, TTP is diffusely distributed in the cytoplasm where it localizes with DCP1 (mRNA-decapping enzyme 1A) in P bodies and in the nucleus [7,14]. P bodies (PBs) are RNP complexes that contain components of the mRNA decay machinery that are present under normal growth conditions and under stress. PBs increase in size and number by fusing with other PBs or even with SGs [14,15].

Under stress conditions, such as exposure to the mitochondrial uncoupler CCCP (carbonyl cyanide m-chlorophenyl hydrazone), nuclear TTP translocates to the cytoplasm to associate with SGs, where it colocalizes with the SG marker TIA1 [7]. TTP assembly into SGs is regulated by post-translational modifications, such as phosphorylation; for example, TTP is phosphorylated in its S52 and S178 residues to form a TTP:14-3-3 complex, which excludes TTP from SGs and inhibits the degradation of ARE-containing transcripts [7]. Diverse kinases phosphorylate TTP, such as MAPKAP kinase-2 (MK2) [7], c-Jun N-terminal kinase (JNK), p38 MAP kinase, and p42 mitogen-activated protein kinase (ERK2) [16].

*C. elegans* has several genes that encode for proteins with zinc finger domains similar to TTP, such as *pie-1*, *pos-1*, *mex-1*, *mex-5*, *mex-6*, and *oma-1/-2* [17–20]*.* In this work, we studied one of the TTP nematode’s homologs, GLA-3, in the *C. elegans* gonad during stress. The *C. elegans* gonad is an excellent model for studying biomolecular condensates *in vivo* because of its vast size and transparency, among other features [21,22]. Previous studies identified SGs assembly in the *C. elegans* gonad during heat shock, starvation, prolonged meiotic arrest, among other conditions [23–26].

By alternative splicing, *gla-3* produces three isoforms, which are expressed in the soma and germline during early embryogenesis, L4 larvae, and the adult stage [27]. *C. elegans* GLA-3 is a novel component of the MAP kinase MPK-1 signaling pathway required for germ-cell survival [27]. *gla-3* loss of function or silencing exerts many effects on the nematode. Among the latter are found progressive loss of motility due to protein degradation in muscle, fertility issues due to defects in meiotic progression, high levels of germ cell apoptosis, and a less severe effect on embryonic lethality [27–29]. Two hybrid and immunoprecipitation assays identified an association between GLA-3 and the MAP kinase MPK-1/ERK that is required for pachytene exit during meiosis [27]. Germ cells in the pachytene region of *gla-3*-mutant animals present a delay in their progression that could be caused by a misregulation of the MAPK signal [27,29]. Despite that the GLA-3 function has been studied in *C. elegans*, we do not yet know its role in the heat shock response in this or other organisms.

Previously, we found that exposing young adult hermaphrodites to up to 6 hr of starvation (bacterial deprivation) or 3 hr of heat shock (31^o^C) led to increased germ-cell apoptosis, and the formation of ribonucleoprotein (RNP) complexes or biomolecular condensates in the gonad’s core or raquis [25,30]. These stress-induced condensates behave similarly to stress granules, as TIAR-1 (the *C. elegans* homolog of TIA-1) associates with them and is required for their formation. Additionally, stress-induced condensates in the *C. elegans* germline form in the presence of puromycin and dissociate upon treatment with ciclohexamide and 1,6-hexanediol [25,26]. However, we also observed that CGH-1, the *C. elegans* homolog of DDX6, a typical processing body protein, associates with these condensates.

Here, we sought to determine whether stress-induced condensates in the *C. elegans* gonad core contain markers of P bodies or stress granules. Using fusion proteins for SG markers TIAR-1 and G3BP-1, as well as the P body markers GLA-3 and CGH-1, we found that condensates formed during heat shock contain both P body and stress granule markers. A similar phenomenon occurs in yeast, where P bodies serve as nucleation sites for stress granule formation. We further investigated the role of GLA-3 in condensate formation during heat shock. We found that *gla-3* mutant animals failed to form condensates. However, when *gla-3* was silenced, the animals still formed heat shock-induced condensates in their gonad core; however, the size and distribution of these condensates were altered, suggesting that GLA-3 is important for their proper formation. Furthermore, we demonstrated that GLA-3 plays an essential role in protecting germ cells during heat shock.

## Materials and methods

### Strains

*C. elegans* strains were maintained at 20°C on NGM-Lite and fed with the *Escherichia coli* strain OP50-1 [31]. The following strains were used: wild type variety Bristol N2, WS2974 *gla-3(ep312)* [27], DG4230 *gla-3a(tn1734[gfp::3xflag::gla-3a])* [32], DG3922 *tiar-1::gfp* [25] and JH3199 *gtbp-1::gfp* [33]. This study does not require an “Ethics Statement” because it does not involve human participants, specimens, or tissue. It does not involve vertebrate animals or cephalopods, vertebrate embryos or tissues, or field research.

### RNA interference

To silence the *gla-3* gene, we obtained the corresponding clone from the RNAi library (OpenBiosystems), and the plasmid pPD129.36 was used as control (EP, empty plasmid) [34]. We used the *E. coli* strain HT115(DE3) to feed animals for RNAi experiments. To induce the production of double-stranded RNA, we followed standard procedures [35]. Briefly, bacterial cultures were grown overnight in LB broth containing 50 μg/ml of ampicillin and 12.5 μg/ml of tetracycline. To induce double-stranded RNA formation, a drop of overnight cultures was seeded onto 60 mm NGM plates supplemented with ampicillin (50 μg/ml), tetracycline (12.5 μg/ml) and IPTG (1 mM). Plates were incubated overnight at room temperature in the dark to allow double-stranded RNA synthesis. L4 larvae were placed onto NGM-lite plates containing induced bacteria and incubated at 20°C for 24 hr for RNA silencing.

### Stress conditions

Synchronized L1 animals were grown at 20°C on NGM-lite plates seeded with indicated bacteria until they were 1-day-old (1-d-old) adults. Then the population was separated into stressed and control groups. For starvation conditions, 1-d-old animals were transferred to NGM-lite plates without bacteria and incubated for up to 6 hr at 20°C. For the control group, animals were kept on NGM-lite plates seeded with indicated bacteria at 20°C. For starvation recovery experiments, animals were transferred into NGM-lite plates seeded with indicated bacteria and kept at 20°C for the desired time after stress. For heat shock treatment, 1-d-old animals were transferred to seeded plates, sealed with Parafilm, and placed in a temperature-controlled water bath at 31°C for 3 or 5 hr, as indicated in each figure legend. The control (no stress) group were kept on seeded plates in the incubator at 20°C. For heat shock recovery experiments, plates were transferred to an incubator and kept at 20°C for the indicated time after stress. After every treatment or recovery time, animals were anesthetized with 3 µM tetramisole, mounted on 2% agarose pads and observed under an epifluorescence Nikon E600 microscope equipped with an AxioCam MRc camera or a confocal Zeiss LSM8001 microscope.

### Immunostaining

To visualize stress granules using CGH-1 as a marker, we performed immunostaing as previously reported by [36]. Briefly, the gonads of 1-d-old animals were dissected, freeze-cracked, and fixed in cold methanol (-20°C) for 1 min. Samples were fixed in a solution containing 3.7% paraformaldehyde, 80 mM HEPES, 1.6 mM MgSO4, and 0.8 mM EGTA dissolved in 1X PBS for 15 min at room temperature. After fixation, samples were washed twice with PBT, and were then blocked in PBT containing 30% normal goat serum (NGS; Sigma-Aldrich, St. Louis, MO) for 30 min. Primary antibody were diluted in PBT with NGS and the incubation was performed overnight at 4°C with rabbit anti-CGH-1 (1:1000) [37], and mouse anti-GFP (1:100; A11120 from Molecular probes, Eugene, OR). Secondary antibody incubations were performed for 3 hr at room temperature using an Alexa Fluor 594-conjugated polyclonal goat anti-rabbit antibody (A-11012; 1:100) and an Alexa Fluor 488-conjugated polyclonal goat anti-mouse antibody (A-11001, 1:1000; Invitrogen, CA). To detect DNA 1ng/μl 4′6′-diamidino-2-phenylindole (DAPI) was used. Vectashield Mounting Medium (Vector Laboratories, Burlingame, CA) was added to avoid photo bleaching before sealing the sample. At least two independent experiments were conducted with n ≥ 20 for each condition and time point. The average percentage of animals with visible granules is depicted in the graphs.

### Image analysis

Microscopy images were obtained using a Zeiss LSM8001 confocal microscope. To achieve high-resolution images suitable for analysis, a pixel size of 2048 x 2048 was used. To quantify condensates in gonads, five consecutive nuclei were analyzed starting from the end of the nuclei transition zone. The first region analyzed included 1–5 near the gonad loop, followed by the region containing nuclei 6–10. Analyses were performed in the gonad raquis to avoid germ granules, which are usually located at the periphery. Condensates were segmented in FIJI using AutoThreshold (IJ-IsoSata) for the green channel (Alexa 488) and Intermodes thresholding for the red channel (Alexa 594). Condensate’s size was quantified using particle analysis, excluding objects smaller than 0.01 µm^2^ to avoid false positives. For co localization analysis, the BIOP JACoP plugin was used, applying the same regions of interest and thresholding settings as in the condensate quantification.

### Quantitation of embryonic lethality after stress

Experiments were performed as previously published by [25]. Hermaphrodites were cultured at 20°C and individually cloned onto plates at the mid-L4 stage. After 18–20 hr, young adult hermaphrodites were transferred to seeded plates, sealed with Parafilm, and incubated in a water bath at 31°C for 3 hr. Control (non-stress) animals remained on NGM-lite plates seeded with OP50−1 at 20°C. Immediately following heat stress, animals were mounted without any anesthetic on 2% agarose pads with M9 and observed under the microscope. The number of embryos in the uterus and fully-grown oocytes (positions –1 to –3) in each gonad arm was recorded for each hermaphrodite. After imaging, animals were returned to NGM-lite seeded plates at 20°C and allowed to lay the same number of embryos previously counted, constituting group I. The hermaphrodites were then transferred to fresh plates and allowed to lay embryos for an additional 12 hr (group II), followed by another transfer for a subsequent 24-hour laying period (group III). Embryonic lethality was assessed as the percentage of embryos that failed to hatch 24 hr after being laid. Parallel, non-stressed control groups were processed identically to determine baseline embryponic lethality.

### Statistical analyses

Statistical analyses are described in the respective figure legends. Data is available at S1 Table.

### AI tool

We used ChatGPT (GPT-4, OpenAI) to assist with language editing, grammar correction, and improving clarity and flow throughout the manuscript and cover letter. All AI-generated suggestions were reviewed, edited, and approved by the authors. No original research data, analysis, or conclusions were generated using the AI tool. The authors take full responsibility for the content of the manuscript.

## Results

### GFP::GLA-3a is expressed in the *C. elegans* germline

**Fig 1:**
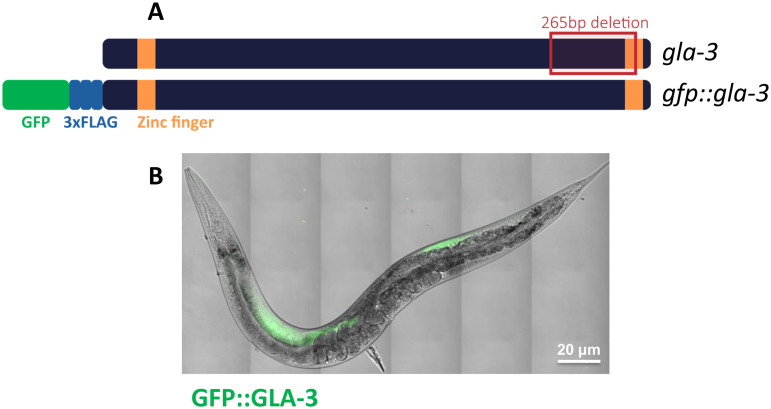
GFP::GLA-3 expression in the adult hermaphrodite. **A)** Scheme of the GLA-3 protein showing two zinc finger domains (orange boxes), the site of the *gla-3(ep312)* mutant deletion (outlined in red) and the GFP and FLAG tags in the *gfp::gla-3* transgene*.* The mutation was generated by EMS, which deleted a 265 bp region and introduced a frameshift at the new junction, affecting the three isoforms and removing part of the last two exons of *gla-3* [27]. The *gfp::gla-3* construct was generated using CRISPR-Cas9 by the Greenstein Laboratory [32]. The GFP is in the N-terminus of the GLA-3 protein (green) and carries a 3xFLAG at the N-terminus (blue). **B)** Merge image of a live adult animal observed in Nomarski and epifluorescence microscopy showing GFP::GLA-3 expression in the gonad under normal growth conditions. Scale bar, 20 µm.

**Fig 2:**
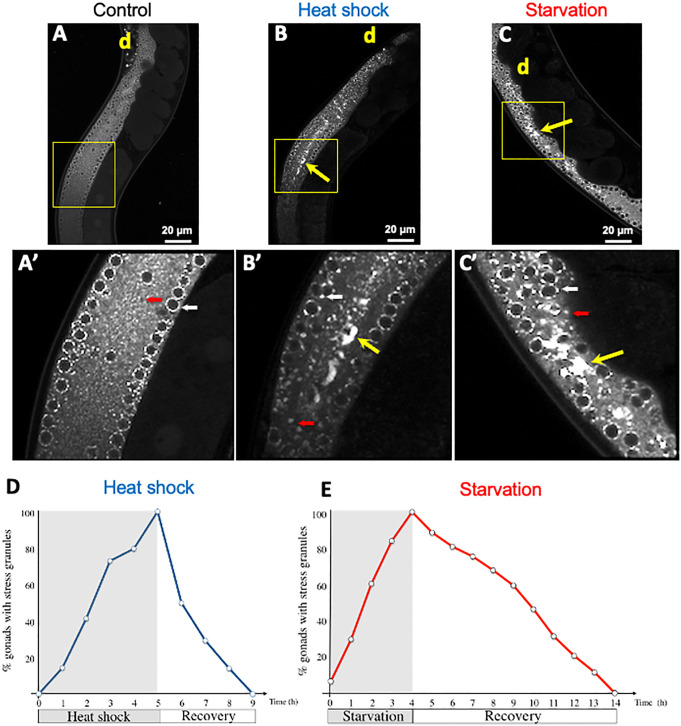
The formation of GFP::GLA-3 condensates during heat shock or starvation is transitory. **(A-C)** Confocal images of *gfp::gla-3* transgenic animals under control **(A)**, heat shock (3 hr 31°C) (B) or 6 hr of bacterial deprivation **(C)**. Details of each image are shown on A’, B’ and C’, respectively (yellow boxes). White arrow point toward perinuclear foci (germ granules), red arrow point toward scattered cytoplasmic GLA-3 condensates (putative storage bodies or P bodies) and yellow arrows point toward gonad core GLA-3 condensates form during stress. d = distal. Scale bars, 20 μm. **(D and E)** Graphs showing the percentage of animals that have GLA-3 granules under indicated conditions. *gfp::gla-3* 1-d-old hermaphrodites were exposed to heat shock (up to 5 hr at 31°C. D, blue line) or starvation (4 hr with no bacteria. E, red line). Animals were observed under the epifluorescence microscope every hour and were scored for the presence of GFP::GLA-3 gonad core condensates during heat shock or starvation exposure and recovery, until condensates were no longer observed. Two independent experiments were conducted under each condition and time point (n = 50). The average percentage of animals with visible granules is depicted in the graphs.

To study the expression of GLA-3 *in vivo*, we used the DG4230 strain previously generated by CRISPR-Cas9 genome editing by the Greenstein Laboratory [32]. This strain expresses *gla-3* isoform a fused to a *gfp* reporter in the amino-terminal and a 3xflag tag; *gfp::3xflag::gla-3a* (https://wormbase.org/species/c_elegans/gene/WBGene00011376#0-9f-10). From this point on, we will refer to this fusion protein as GFP::GLA-3 (Fig 1A)*.* Under normal growth conditions, we observed that GFP::GLA-3 expression in the distal gonad of adult hermaphrodite animals (Fig 1B). GFP::GLA-3 is restricted to the germline from the early larval stage through adulthood (S1 Fig). GFP::GLA-3 expression is mainly observed in germ cell cytoplasm and in perinuclear foci that resemble germ granules (S1 Fig; yellow arrows). In the adult hermaphrodite gonad, GFP::GLA-3 expression is restricted to the distal gonad tip prior to the bend region of the gonad (Fig 2A).

### GFP::GLA-3 associates with condensates during heat shock and starvation

To study GFP::GLA-3 expression, we subjected 1-d-old *gfp::gla-3* animals to 31°C for 3 hr (heat shock), 6 hr of starvation (no bacteria) or kept them under control conditions (20°C with food *at libitum*). Control, heat-shocked, and starved animals were mounted for observation under a confocal microscope. Control animals showed GFP::GLA-3 expression in the distal gonad in the germ cells’ cytoplasm and in perinuclear granules that resemble germ granules (white arrow Fig 2A and 2A’). We also observed GFP::GLA-3 expression in germ cell’s cytoplasm as a scattered pattern (red arrow Fig 2A and 2A’) that could be putative storage bodies or P bodies [36,37]. Heat-shocked animals exhibited larger and fewer perinuclear GLA-3 condensates (white small arrow), larger scattered GLA-3 condensates (red arrow), and large GFP::GLA-3 condensates in the middle of the gonad core (yellow arrow) (Fig 2B and 2B’). Starved animals showed perinuclear GLA-3 condensates (white arrow), scattered punctuated GLA-3 condensates in the gonad core (red arrow), and large GLA-3 condensates in the middle of the gonad core (yellow arrow) (Fig 2C and 2C’).

A feature of stress granules is that they are transitory [6,25]; therefore, we examined the assembly and disassembly kinetics of GLA-3 gonad core condensates. For this, *gfp::gla-3* animals were subjected to heat shock or starvation conditions, until all animals had formed GLA-3 condensates, 5 hr for heat shock and 4 h for starvation, respectively (Fig 2D and 2E). Animals were mounted every hour under the microscope to quantify the percentage of gonads that showed condensate formation. GLA-3 condensates, formed under heat-shock conditions, disassemble gradually in 4 hr; in contrast to those formed under starvation, which lasted for 10 hr. Our data showed that the association of GFP::GLA-3 to gonad core condensates under tested conditions is reversible. For the purposes of this study, we will focus solely on the role of GLA-3 in the heat shock response.

### Stress granules and P bodies colocalize during heat shock

**Fig 3:**
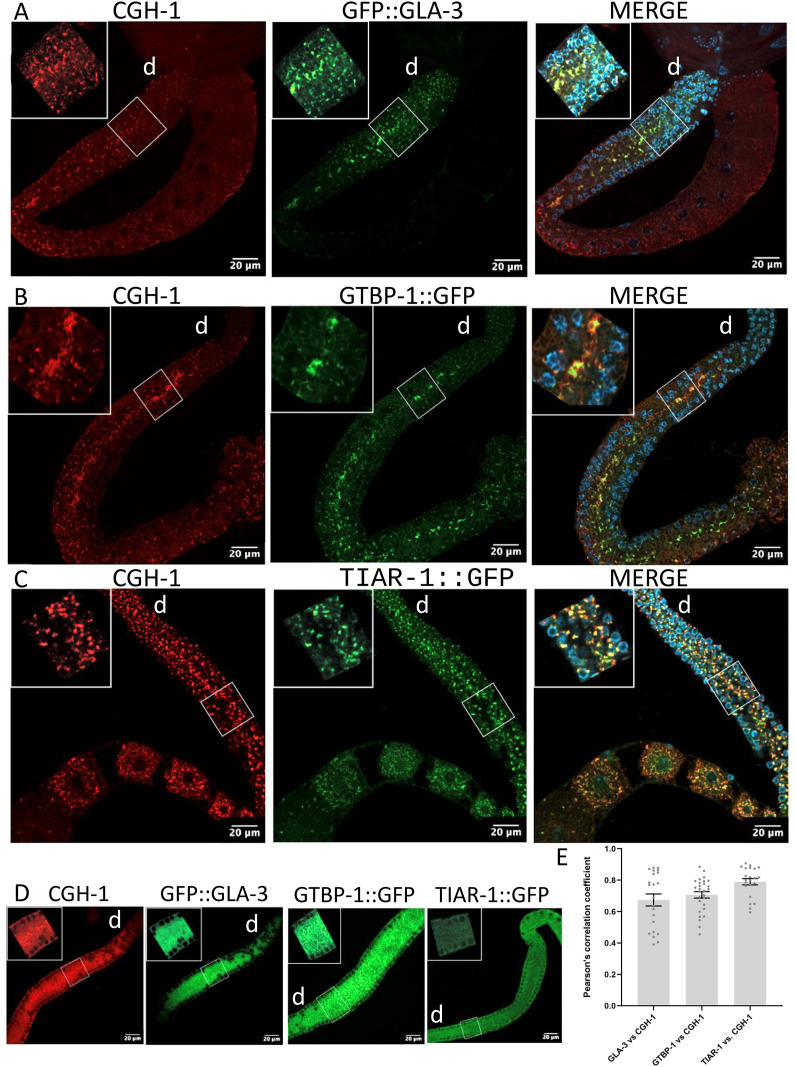
P bodies and stress granules colocalize during heat shock in the gonad core. 1-d-old hermaphrodites from indicated genotypes were subjected heat shock (31°C for 3 hr) or kept in control conditions (20°C). After treatment, gonads were dissected, fixed, and co-stained using GFP and CGH-1 antibodies and DAPI to visualize DNA. A-D). Confocal images of dissected gonads in control (D) or heat shock conditions **(A-C)**. Details of each picture are shown in white boxes. Scale bars, 20 μm. d = distal. **E)** The Pearson’s correlation coefficient indicates a high correlation of CGH-1 with GLA-3 (0.67 ± 0.038; n = 22), GTBP-1 (0.70 ± 0.10, n = 28), and TIAR-1 (0.78 ± 0.02; n = 22).

Previously, we reported that condensates forming in the *C. elegans* gonad core under heat shock and starvation conditions share properties with mammalian stress granules [25]. To test whether GFP::GLA-3 associates with stress granules or P bodies, we performed colocalization experiments using two transgenic lines expressing typical stress granule markers, TIAR-1::GFP and GTBP-1::GFP, as well as an antibody against the RNA helicase CGH-1, a characteristic P body marker and the *C. elegans* homolog of DDX6 [25,33,37]. 1-d-old animals were exposed to heat shock at 31°C for 3 hr. After the heat shock, gonads were dissected and processed for immunostaining using antibodies against GFP and CGH-1 (see Materials and Methods). Under control conditions, CGH-1 is dispersed in the cytoplasm and associates with small foci resembling P bodies (Fig 3D). During heat shock, CGH-1 associates with larger condensates (Fig 3A–3C).

Under control conditions, GFP::GLA-3, GTBP-1::GFP and TIAR-1::GFP are dispersed in the cytoplasm (Fig 3D). In contrast, under heat shock, all three reporter proteins associate with condensates (Fig 3A–3C). Unexpectedly, we observed a high degree of colocalization between CGH-1 and GFP::GLA-3 as well as TIAR-1::GFP and GTBP-1::GFP (Fig 3A–3E). Particularly, CGH-1 colocalization was higher with TIAR-1::GFP, suggesting that during heat shock, P bodies and stress granules merge in the *C. elegans* gonad. Fron now on, we will refer to heat shock-induced granules as stress granules.

### GLA-3 is important for stress granules formation during heat shock

**Fig 4:**
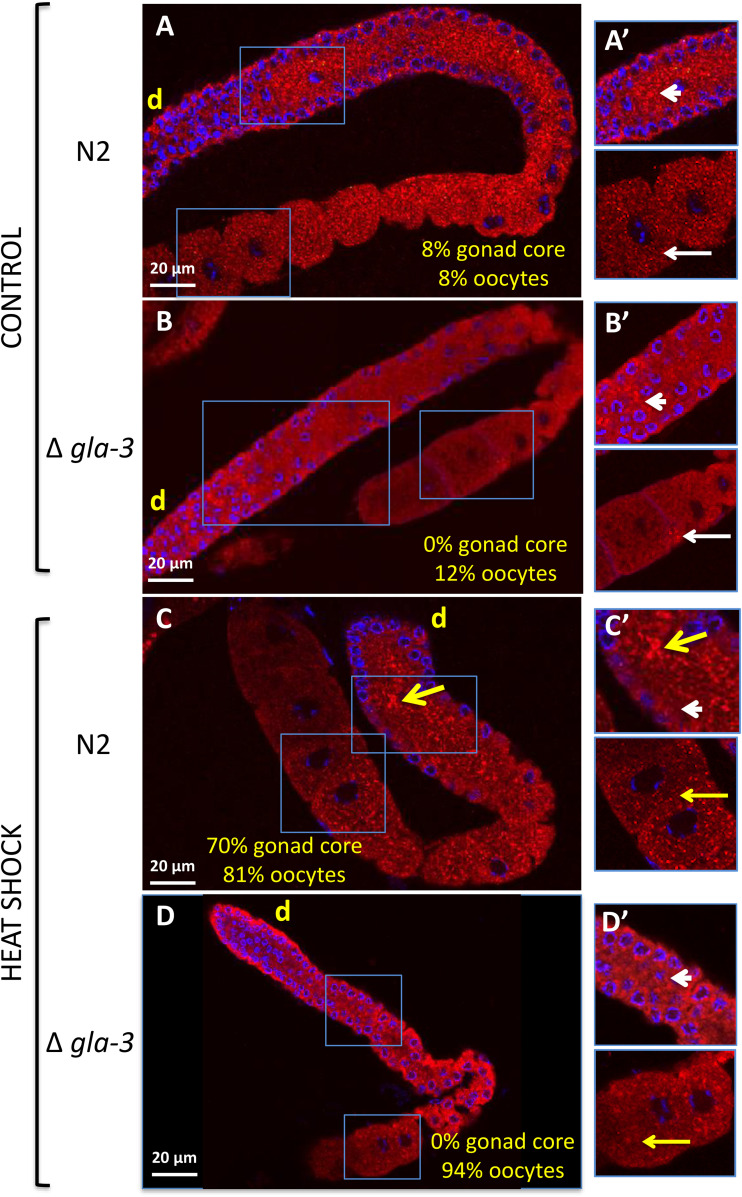
*gla-3* mutant animals are unable to form stress granules under heat shock. A-D) Wild type (N2) (A and C) and *gla-3(ep312)* (B and D) 1-d-old animals were maintained under control conditions (20°C) (A and B) or exposed for 3 hr to 31°C to induce heat shock (C and D). After heat shock, the gonads were extruded, fixed, and stained with a rabbit anti-CGH antibody (red) and DAPI (blue). Samples were mounted under the confocal microscopy for observation and quantification of animals showing stress granules. The percentage of gonads that presented CGH-1 stress granules in the gonad core or oocytes is indicated in each panel. A’-D’) Details of each gonad are shown at the right for each picture (blue boxes). Yellow arrows point toward heat shock-induced CGH-1 granules in the gonad core (thick yellow arrows) or oocytes (thin yellow arrows). White arrowheads (gonad core) and thin white arrows (oocytes) point toward CGH-1 condensates present in control conditions (storage bodies). d = distal. At least two independent experiments were conducted under each condition (n = 50).

TTP, the GLA-3 homolog in mammals, is involved in stress-granule assembly [7]. We found that GLA-3 is also required for stress granules formation during heat shock in the *C. elegans* gonad. To carry out these experiments, we used an antibody against CGH-1 as a granule marker. Under control conditions, CGH-1 is found in the germ-cell’s cytoplasm in a punctuated pattern known as storage bodies or P bodies (Fig 4A, 4A’). Under heat shock conditions, CGH-1 accumulates in granules in the gonad core and the oocytes (Fig 4C, 4C’) [25]. *N2 and gla-3(ep312)* 1-d-old animals were subjected to control conditions (20°C) or heat-shocked (3 hr at 31°C). After stress, the gonads of control and treated animals were dissected, fixed and stained with a rabbit anti-CGH-1 antibody and DAPI to detect DNA. In heat-shocked wild type animals, CGH-1 accumulated in SGs in the gonad core (70%) and oocytes (81%) of the gonads observed (Fig 4C and 4C’). In contrast, none of the gonads from *gla-3(ep312)* animals formed stress granules under control or heat shock conditions (Fig 4B, 4B’, 4D and 4D’). A small percentage of *gla-3(ep312)* gonads revealed CGH-1 granules in oocytes under control conditions (12%), while the majority demonstrated SGs in the oocytes under heat shock (94%) (Fig 4D and 4D’). We noticed that the small condensates known as storage granules or P bodies are diminished in number in *gla-3* animals under both control and heat shock conditions (Fig 4A’, 4B’, 4C’ and 4D’). Our data shows that GLA-3 is important for CGH-1 association to P bodies and SGs during heat shock.

**Fig 5:**
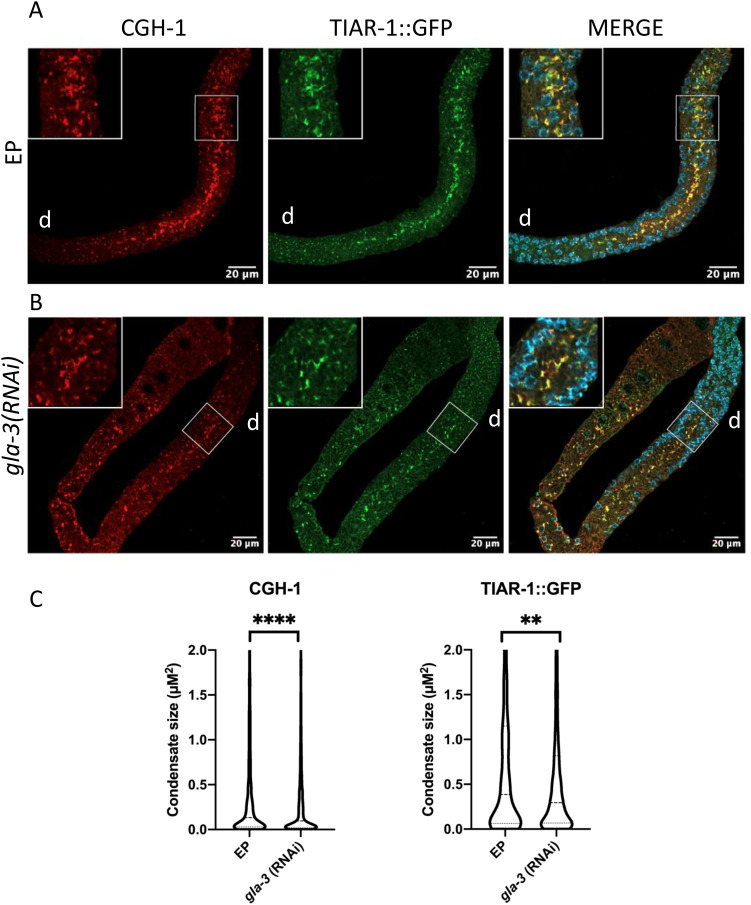
*gla-3* silencing diminishes the size and distribution of CGH-1 and TIAR-1 condensates in response to heat shock. Synchronized L1 *tiar-1::gfp* animals were fed bacteria expressing *gla-3* dsRNA or an empty plasmid (EP) as control. EP and *gla-3(RNAi)* 1-d-old hermaphrodites were kept at 20°C or exposed to heat-shock (31°C for 3 hr). After treatment, the gonads were dissected, fixed and co-stained with DAPI to visualize DNA and antibodies against CGH-1 and GFP. A and B) Confocal images of gonads from EP (A) or *gla-3(RNAi)* fed animals (B) are shown. Details of each picture are shown in white boxes. d = distal. C) Graphs showing condensate’ size quantification in a single Z-slice of the indicated genetic backgrounds in heat shock exposed animals. Condensate’ size was only measure in the central gonad core, avoiding germ granules, and in the regions depicted in the white boxes. The size of condensates might vary along the gonad. Dashed lines within each violin plot indicate the median size. For EP fed animals, CGH-1 condensates have a median size of 0.1330 ± 0.03 µM, while condensates of *gla-3(RNAi)* measure 0.095 ± 0.02 µM. TIAR-1::GFP condensates in EP animals have a median size of 0.39 ± 0.04 µM, compared to 0.30 ± 0.03 µM in *gla-3(RNAi)* animals. Unpaired t-tests were performed to determine statistical significance. *gla-3*(RNAi) n = 31 EP n = 25 (**) P= < 0.01 (****) P= < 0.0001.

To test the effect of silencing *gla-3* on CGH-1 and TIAR-1 expression under heat shock conditions, we performed RNAi of *gla-3* in *tiar-1::gfp* transgenic animals to visualize stress granules and used an antibody against CGH-1 as a P body marker. As a control for the RNAi experiments, we used an empty vector plasmid (EP) [35]. We tested *gla-3* silencing by performing RNAi in the *gfp:gla-3* animals, and the silencing efficiency was 96% (27 out of 28 animals showed no or very low *gfp::gla-3* expression). We found that CGH-1 and TIAR-1::GFP condensates still formed after *gla-3* silencing, however they looked more dispersed and smaller (Fig 5A and 5B). CGH-1 condensates had a mean size of 0.59 µm ± 0.03 µm in EP heat shock, whereas in *gla-3(RNAi)* gonads of animals exposed to the same conditions, their mean size was slightly reduced to 0.45 ± 0.02 µM (Fig 5C). Similarly in EP of heat shock animals, TIAR-1::GFP condensates had a mean size of 0.84 ± 0.04 µm and reduced to 0.68 µm ± 0.03 µm in *gla-3(RNAi)* animals (Fig 4C). Our data suggest that GLA-3 contributes to the formation of P bodies and SGs during heat shock.

### GLA-3 protects germ cells from heat shock

**Fig 6:**
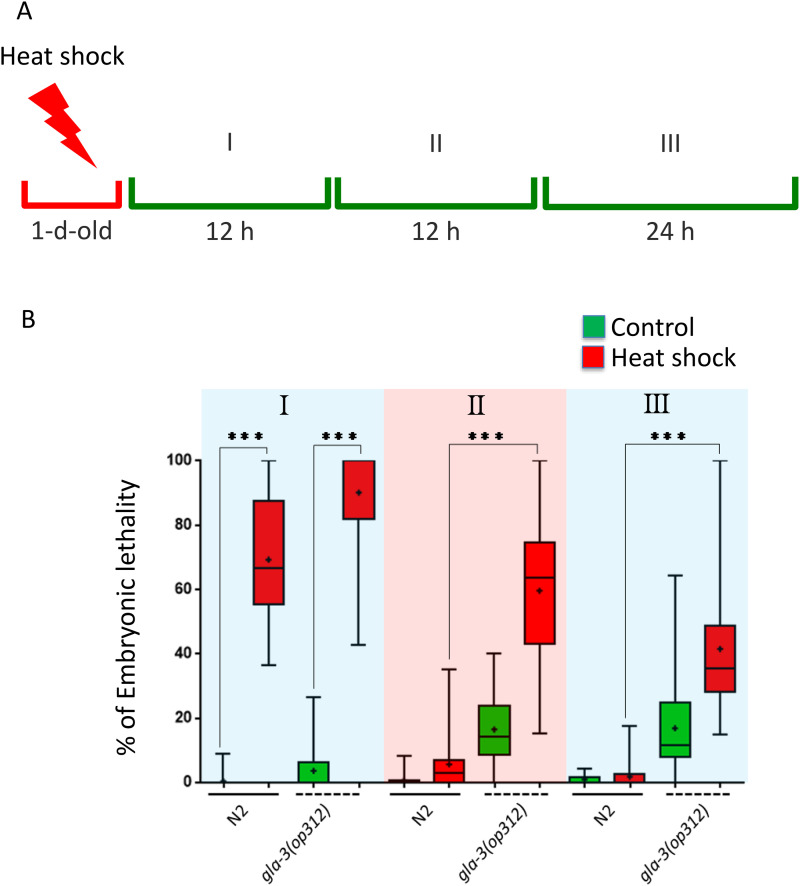
GLA-3 protects germ cells from heat shock. **A)** Scheme representing how the groups of embryos were assigned. 1-d-old N2 and *gla-3(ep312)* animals were either exposed to a 3 hr heat shock at 31°C or maintained at 20°C as controls (not shown for simplicity). Following heat shock, animals were mounted without anesthetic on 2% agarose pads with M9 and examined under a microscope. The number of embryos and mature oocytes (positions −1 to −3) in each gonad arm was recorded for each hermaphrodite. Animals were returned to 20°C and allowed to lay the same number of embryos previously counted, constituting group I (12 hr). Group II consisted of embryos laid 12 hr after Group I, and Group III included embryos laid 24 hr later of Group **III.** Embryonic lethality was assessed 24 hr after each transfer by counting the number of unhatched embryos 24 hr after the parental hermaphrodite was removed. **B)** The Graph show the data obtained from two independent replicates (n = 20). Green bars represent animals maintained at 20°C (control), while red bars represent those subjected to heat shock. The boxes represent the interquartile range (IQR) from 25%−75%, and the bars extend from the minimum to the maximum value. The three asterisks indicate significance with a value of p < 0.001. The statistical model employed was one-way ANOVA with the Bonferroni multiple comparisons test.

To study the role of GLA-3 in the heat shock response in *C. elegans*, we subjected 1-d-old adults to 31°C and followed animal’s progeny over several hours afterward to quantify embryonic lethality. After heat shock, the animals’ progeny was followed for 48 hr (see Materials and Methods). Wild type animals showed high embryonic lethality in the first group and much lower in the group II, while the group III exhibited nearly no embryonic lethality (Fig 6). In contrast, *gla-3(op321)* animals continued to show high levels of embryonic lethality even several hours after the heat shock was performed in groups II and III (Fig 6). These data suggest that GLA-3 is important for protecting germ cells from heat shock.

## Discussion

The TTP family of proteins has been extensively studied *in vitro*; however, its role in the germline and under stress conditions remains poorly understood. Here, we show that the TTP homolog in *C. elegans*, GLA-3, plays an important role in germ cells. Germ cells from *gla-3* mutant animals are more sensitive to heat shock and fail to form P bodies and stress granules. Silencing of *gla-3* leads to a reduction in both the number and size of condensates during heat shock. We also show that, during heat shock, condensates are positive for CGH-1/DDX6, TIAR-1/TIA, and G3BP-1/G3BP, demonstrating that P bodies and stress granules colocalize under these conditions in the *C. elegans* gonad. Our results indicate that GLA-3 retains its role in *C. elegans* as an important regulator of mRNA expression and plays an important role in the heat shock response.

### P bodies and stress granules merge during heat shock in the *C. elegans* germline

Germ cells have several types of biomolecular condensates that control mRNA expression during gametogenesis [2,22,38]. Here, we demonstrate that GLA-3 associates with germ granules in germ cells, as well as with condensates in the gonad that are positive for both P bodies and stress granules markers.

*C. elegans* germ cells possess condensates similar to P bodies that have been observed using proteins such as CGH-1, CAR-1, DCP-2, MEG-1, MEG-2 and TIS11-family RNA-binding protein POS-1 among others [23,36,37,39,40]. Here, we show that in control conditions, the GLA-3 protein is dispersed in the cytoplasm and associates with small granules within the germ cells that resemble P bodies. Several lines of evidence have demonstrated that TTP associates with P bodies in mammals. For example, TTP binds to ARE-containing mRNAs to promote their decay and drive them to P bodies [41]. Additionally, when mRNA decay is inefficient, TTP sequesters ARE-mRNA in PB. Furthermore, when enzymes related to mRNA degradation, such as XRN1 or DCP2, are knocked down, ARE containing mRNAs accumulated in P bodies along with TTP [42]. TTP can also trigger PBs formation when cells are treated with cycloheximide, which normally disrupts PB formation due to translational arrest [42]. Due to the association of GLA-3 with PB-like condensates, it is possible that GLA-3 might play similar roles to those of TTP in the nematode’s germline. Whether GLA-3 promotes mRNA degradation of ARE-containing mRNAs like its mammalian homolog remains to be elucidated.

Upon stress, *C. elegans* germ cells trigger the formation of large condensates that share many features of mammalian stress granules (reviewed by [22]). For example: 1) gonad-stress granules have several conserved stress-granule markers such as TIAR-1, CGH-1 (an RNA helicase) [23,25,43], GTBP-1 [33] and PAB-1 [23]; 2) Similar to SGs in mammals, germline SGs are formed in response to translational arrest [25]; 3) they disassembled in the presence of cycloheximide and assembled in the presence of puromycin [25], and 4) similar to SGs, gonad-stress granules formation is transitory [25].

Intriguingly, gonad’s stress granules in *C. elegans* also have some markers of P bodies, such as DCP-2 [23], CAR-1/RAP55 [23] and CGH-1/DDX6 [23,25]. Here, we demonstrated that in the *C. elegans* gonad, during heat shock, stress granules and P bodies co-localize. CGH-1 and GLA-3 showed the lower degree of colocalization compared to CGH-1 with GTBP-1 and TIAR-1 (Fig 3). These results demonstrate that stress granules and P bodies in *C. elegans* are closely related and, at the same time, are similar to their counterparts in yeast and mammals [44,45].

The way in which different biomolecular condensates limit their formation and interaction is still an open question that we are starting to comprehend. In yeast, during glucose deprivation, PBs assemble first followed by SGs and the preexistence of PBs enhances the formation of SGs. On the contrary, the assembly of PBs does not require SGs preexistence. Interesting in yeast, once formed, stress granules and P bodies remained mixed during stress [44]. In mammalian cells, SGs form independently of PBs upon arsenite stress [44] and are typically distinct compartments that remained separated, but come into contact through a process known as docking [14]. Usually, PBs and SGs are highly motile, but when they dock, they appear less motile presumably facilitating the exchange of components. In mammals, it has been recently shown that the DEAD RNA helicase DDX6 avoids the mixing of SGs components with PBs in a non RNA binding and ATPase activity manner [46]. When DDX6 is absent, SGs grow bigger than PBs, and docking of small PBs with SGs is prolonged. Similarly, the overexpression of TTP in mammals induces the fusion of P bodies and stress granules [14,47], and smaller PBs dock to SGs. Like DDX6, TTP must compete for the mRNAs present in both types of condensates. Here we found that silencing GLA-3/TTP diminished the size of both SGs and P bodies; however, these condensates remained mixed (Fig 5).

Although stress granules observed in the *C. elegans* gonad core and oocytes associate with the same proteins, they diverge in some aspects. Particularly, gonad core stress granules require TIAR-1 or GLA-3 for their formation, while oocyte-stress granules do not ([25] and this work, respectively). Oocyte condensates are also observed in hermaphrodites that do not have sperm; this condition, known as arrested oogenesis, is present in old animals (more than 3-d-old) or some genetic backgrounds that feminized the germline [23]. Large condensates observed in arrested oocytes showed the same markers as PB, and SGs in addition to MEX-3, an RNA binding protein important for germline and embryonic development [48]. However, large condensates in arrested oocytes appear to have liquid-gel consistency because they do not reconstitute after photobleaching as fast as condensates observed in the pachytene germ cells [49,50].

P granules are generically known in other organisms as germ granules, which are present in most germ cells. In 1998, Brangwynne et al. demonstrated that P granules showed liquid properties [1]. This discovery was a landmark for the field of liquid-liquid phase separation. Germ granules are sites of maternal inheritance, mRNA sorting, post-transcriptional regulation, and small RNA biogenesis (recently reviewed by [3]). The association of GLA-3 with germ granules opens the possibility that this protein plays an important role in any of these functions. Interestingly, the GFP::GLA-3 is expressed mainly in germ cells that are undergoing pachytene; particularly in this region, transcription is very active. It is possible that GLA-3 regulates the expression of maternal mRNAs that are transcribed in this region.

### The TTP family of proteins plays an important role in stress granules formation

There are some proteins that participate in the condensation of granules in the *C. elegans* germline, but much remains to be learned about this mechanism. Germ cell P granule nucleation is the best-characterized in *C. elegans*, and mainly requires the RNA binding proteins PGL-1, −2, and −3 and the DEAD box RNA helicases GLH-1 and GLH-4 (reviewed by [51]). PGL and GLH proteins possess intrinsically disordered or low complexity domains that bestow on P granules a liquid-like behavior [52]. During late oogenesis and early embryogenesis, P granules detach from the nuclear pores and get surrounded by the intrinsically disordered proteins MEG-3 and MEG-4 conferring them a gel-like consistency that bestows them more resistant [53]. Despite that MEG-3/MEG-4 are present in oocytes, surprisingly, these proteins do not localize to all large oocyte granules, and they are not necessary for PGL-1, CGH-1, or MEX-3 association to these RNPs [50].

A key player in the condensation of large oocyte granules during arrest oogenesis conditions is the PUF family of translational repressors [49]. The PUF proteins are necessary for the condensation of CAR-1 or the deadenylase CCF-1 into large oocyte granules. Particularly, PUF-5 contributes to the condensation of MEX-3 and MEG-3 into large granules in arrested oocytes; however, it is not important for PGL-1 condensation into these particles [50]. Intriguingly, neither MEX-3, TIAR-1, nor GLA-3 play a role in oocyte SG formation in arrested oocytes or under stress conditions ([25,50] and this study). It is possible that PUF-5 or other members of its family could play a role in oocyte SG formation observed under other stress conditions, such as starvation and heat shock.

The DEAD box RNA helicase CGH-1 plays an important role by maintaining condensates in the gonad core and arrested oocytes in a “liquid-gel”-like consistency. When *cgh-1* is absent, condensates containing CAR-1, PAB-1, MEX-3 and CEY-3 in the gonad acquire a square-sheet consistency that does not reconstitute after photobleaching, suggesting a transition to a more solid-like phase [49,54,55].

The role of TTP in stress-granule formation has been described in mammals. The overexpression of TTP induces stress-granule formation even in the absence of stress [7]. However, the association of TTP with stress granules in mammals depends on the specific condition being tested. For instance, oxidative stress induced by FCCP localizes TTP to stress granules; in contrast, another type of oxidative stress induced by arsenite excludes TTP from these condensates [7]. TTP exclusion of SG during arsenite exposure is due to the activation of the p38-MAPK/MK2 kinase cascade, which triggers TTP phosphorylation, disabling it from associating with these condensates [7]. Here we show that the role of GLA-3 in stress-granule formation during stress is conserved in *C. elegans*; notwithstanding this, further studies are required to understand exactly how this family of proteins can influence the formation of these condensates

What are the consequences of a deficiency in condensate formation for an organism? This is an intriguing and fascinating question that remains to be fully elucidated. However, it is not easy to answer because most of the proteins that contribute to condensate formation play multiple roles in RNA regulation; therefore, discerning between their functions inside and outside of a condensate is by no means straightforward. Nevertheless, there is some light that point toward the role of condensates in the *C. elegans* germline. For example, when germ-granule nucleators such as *pgl-1* and *glh-1/-4* are absent animals exhibit sterility [51]. In other cases, the consequence is subtler, such as in *meg-3* and *meg-4* mutant animals that develop a normal adult germline, but that are unable to carry on normal miRNAs biogenesis, which results in sterility over several generations [53]. Silencing genes that participate in MEX-3-granule assembly results in embryonic lethality [56]. Lacking TIAR-1 and GLA-3 proteins affects germ-cell quality when exposed to heat shock ([25] and this work).

In an attempt to answer this question, we disrupted the prion-like domain of TIAR-1 in *C. elegans* [26]. Prion-like domains are important for condensate formation [57]. We found that SGs in the *C. elegans* gonad still formed, although their consistency shifted toward a more liquid state. TIAR-1 prion-domain mutant animals continue to exhibit low fertility but show increased embryonic lethality, suggesting that the role of this protein in condensates may be critical for these functions. Understanding the role of condensates in living organisms requires the use of specific assays, which underscore the importance of developing whole-animal models for studying this phenomenon.

## Supporting information

S1 Fig GFP::GLA-3 expression is restricted to the germline. A-D) Live animals, expressing a GFP::GLA-3 transgene at the indicated larval stages, were anesthetized and observed under confocal microscopy. A’-D’) Details of each gonad are shown at the right (yellow boxes). Arrows point toward germ cells’ perinuclear foci. h = head and t = tail. Scale bar = 100 μm.
(TIFF)

S1 Table Data used for the analyses. Each folder contains the data used to generate the graphs associated with each listed figure.
(XLSX)
