# Supplementary figures and images for: GLA-3 mediates the heat shock response in Caenorhabditis elegans germ cells: A key role for the tristetraprolin (TTP) family
Source: PLoS One. 2026 Jan 2;21(1):e0312069. doi: 10.1371/journal.pone.0312069 (PMC12758685; doi:10.1371/journal.pone.0312069)

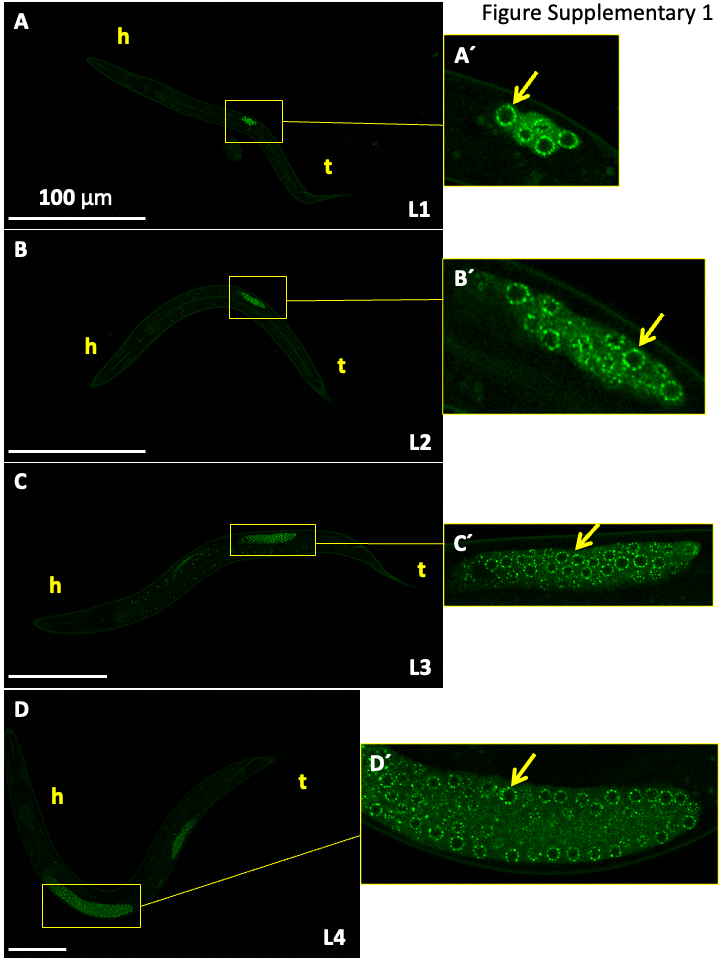

Supplement: S1 Fig — A-D) Live animals, expressing a GFP::GLA-3 transgene at the indicated larval stages, were anesthetized and observed under confocal microscopy. A’-D’) Details of each gonad are shown at the right (yellow boxes). Arrows point toward germ cells’ perinuclear foci. h = head and t = tail. Scale bar = 100 μm. (TIFF) [file pone.0312069.s001.tiff]
